# THE HOPES AND FEARS OF OLDER ADULT, LONG-TERM CANCER SURVIVORS: RACIAL AND GENDER DIFFERENCES

**DOI:** 10.1093/geroni/igac059.2244

**Published:** 2022-12-20

**Authors:** Gary Deimling, Spencier Ciaralli, Dyanna Burnham, Colleen Kavanagh

**Affiliations:** Case Western Reserve University, Cleveland, Ohio, United States; Augustana University, Sioux Falls, South Dakota, United States; Case Western Reserve University, Cleveland, Ohio, United States; Case Western Reserve University, Cleveland, Ohio, United States

## Abstract

Understanding the impact of cancer on the hopes and fears of older adult (age 60+), long-term (5 years +) survivors is important for assessing their quality of life after cancer. Prior quantitative research has shown the important role that cancer plays in the health and psycho-social well-being of survivors in the years and even decades after diagnosis and treatment. This presentation extends that research by examining both quantitative data and survivors’ narratives revealing important issues survivors face such as altered sense of self/identity, feelings of social isolation, key sources of social support as well as cancer and other health-related concerns. These are examined in the context of broader existential issues related to their hopes and fears for the future and differences by race, gender and cancer type are also presented. The data analyzed are from an NCI funded, 10-year longitudinal study based on in-person interviews with 321 survivors of breast, colorectal and prostate cancer to document how cancer-related factors have affected their aspirations for the future. The findings are discussed in the context of how the issues that survivors identified as affecting their hopes and fears may be integrated into the dialogue clinical staff can have with older survivors and their families as part of after-cancer care.

